# Enhanced small neutral but not branched chain amino acid transport after epigenetic sodium coupled neutral amino acid transporter‐2 (SNAT2) cDNA expression in myoblasts

**DOI:** 10.1002/jcsm.12707

**Published:** 2021-05-13

**Authors:** Timothy Pearson, Oskar Wendowski, Penny P. Powell

**Affiliations:** ^1^ Biomedical Research Centre, Norwich Medical School University of East Anglia Norwich UK

**Keywords:** Amino acid transport, Muscle, SNAT2, LAT2, Ageing

## Abstract

**Background:**

Skeletal muscle mass and function are partly maintained by the supply of amino acids, altered amino acid transport is an important cause of frailty that can lead to decreased independence with increasing age and slow trauma recovery. The system‐A sodium coupled neutral amino acid transporter (SNAT)‐2 coded by gene family SLC38A2 generates a 506 amino acid 56 kDa protein that is an important transporter of amino acids in skeletal muscle. Ageing is associated with a decrease in expression of SNAT2 transporters.

**Methods:**

In this study, we used the C2C12 cell line, using myoblast cells and cells differentiated into myotubes. We investigated if the expression of SNAT2 DNA would enhance intracellular amino acid levels and increase their availability for protein synthesis.

**Results:**

In control myoblasts and myotubes, we found significantly decreased expression of SNAT2 (6.5× decrease, *n* = 4 per group, *P* < 0.05) in myotubes than found in myoblasts. After transfection with a SNAT2‐eGFP cDNA plasmid, C2C12 myoblasts significantly increased perinuclear punctate SNAT2‐eGFP expression that persisted and was more cytoplasmic after differentiation into myotubes. Interestingly, transfected cells were significantly more responsive to the hormone 5α‐dihydrotestosterone (DHT, 4.5 nM, by 1.6×, *n* = 3 per group, *P* < 0.04). Starvation significantly enhanced the amino acid C^14^‐MeAIB transport (1.7×, *n* = 3 per group, *P* < 0.05) indicating increased function of SNAT2. Inhibiting SNAT2 with high concentrations of MeAIB (3.3 or 5 mM) significantly reduced C^14^‐Isoleucine transport by L‐type amino acid transporter (LAT2, 52.8% and 77%, respectively, *n* = 3 per group, *P* < 0.05). However, there was no increase in the LAT2 transport of C^14^‐isoleucine detectable in SNAT2‐eGFP transfected cells after DHT (4.5 nM) exposure. This indicated that small amino acid availability was not rate limiting to LAT2 function in myoblasts.

**Conclusions:**

Overall, these data show that transfection of SNAT2‐eGFP expression enhanced its function following starvation and treatment with physiological levels of DHT. Enhanced SNAT2 expression in muscle cells offers a viable epigenetic target in pathological conditions associated with altered amino acid transport.

## Introduction

Skeletal muscle is a major site of metabolic activity and is the most abundant tissue in the human body.^1^ Studies of humans indicate that by the age of 70, there is a ~25–30% reduction in the cross‐sectional area of skeletal muscle and a decline in muscle strength by ~30–40%.^2^ They have further shown a decrease in protein synthesis with ageing; however, the anabolic effect of amino acids is intact in elderly muscle^3^ and argues an imbalance between protein synthesis and breakdown that implies that altered amino acid transport into skeletal muscle may be a contributing factor. Age‐related muscle atrophy and weakness is characterized both by the loss of lean muscle mass and reduced skeletal muscle strength and is a major contributor to frailty and loss of independence in older people.^4^, ^5^ Muscle maintenance and homeostasis has a complex aetiology and the primary biochemical and molecular mechanisms underlying the processes have not been fully identified. Possible causes that have been linked to compromised muscle homeostasis are aberrant ROS production,^6^ changes in amino‐acid transport,^7^ loss of motor units,^8^, ^9^ altered hormone levels,^10^ changes in satellite cell number,^11^ changes in physical activity,^12^ and chronic inflammation.^13^ What is clear is that fast twitch (Type II) glycolytic muscle fibres are more susceptible to ageing associated muscle deficits than slow twitch (Type I) oxidative muscle fibres.^14^

Transport of amino acids into skeletal muscle is achieved through transporters on the sarcolemma, the two most usually found expressed in skeletal muscle being the sodium‐coupled neutral amino acid transporter (SNAT)‐2 and the sodium independent L‐type amino acid transporter (LAT)‐2.^15^ Importantly, these two transporters can work together to transport different amino acid types into muscle cells or fibres. SNAT2 transports small neutral amino acids such as glutamine, glycine, and alanine with one sodium ion into muscle fibres that can then be exchanged by the anti‐porter LAT2^16^ for larger (more difficult to transport) branched chain amino acids, such as isoleucine, valine, and leucine, that are essential for protein synthesis. The LAT2 substrate leucine has been found to be anabolic^17^ compared to valine and isoleucine. Reports indicate decreased expression of SNAT2 protein in old as compared to younger skeletal muscle, an observation more pronounced in fast twitch than slow twitch skeletal muscle.^18^ There was no change in mRNA for SNAT2 (or LAT2), although increased peEF2 (eukaryotic elongation factor‐2) was found in fast twitch muscle fibres that may explain the tissue heterogeneity.^18^ Therefore, one hypothesis that may contribute to pathological conditions in muscle involving perturbed amino acid transport is that reduced SNAT2 expression could limit the availability of small amino acids, for protein synthesis, thereafter also leading to reduced transport by LAT2 of branched chain amino acids essential for protein synthesis. SNAT2 expression has been shown to be increased in response to a DHT treatment,^18^ increasing from internal sources and not by transcription.^19^ Taken together, these observations make SNAT2 a possible target for intervention to maintain amino acid transport to mitigate conditions that could compromise muscle protein homeostasis.

We used the C2C12 cell line as it is a myogenic precursor cell that can be differentiated into mature myotubes.^20^, ^21^ We expressed a GFP tagged SNAT2 cDNA, transfected it into myoblasts and investigated uptake by SNAT2 and LAT2 of amino acid substrates (i.e. C^14^‐α‐(methylamino)isobutyric acid (MeAIB) and C^14^‐isoleucine, respectively) to determine effects on transporter function and sensitivity to physiological stimuli, such as the steroid 5α‐dihydrotestosterone (DHT) and starvation.

## Materials and methods

### Cell culture

The cell line C2C12 (CRL‐1772) is an immortal line of mouse skeletal myoblasts originally derived from the satellite cells of a C3H mouse donor after an injury of the animal's thigh.^22^ Cells were used up to passage 3 and grown in DMEM (Invitogen UK) with 10% fetal bovine serum (FBS, Sigma‐Aldrich, Dorsett, UK), 2 mM l‐glutamine (Sigma‐Aldrich) and 1% penicillin/streptomycin (Invitrogen, UK). Cells were split at ~70% confluence, and myogenic differentiation was induced in DMEM with 2% horse serum supplemented with 2 mM l‐glutamine and 1% penicillin/streptomycin to initiate terminally differentiated myotubes^23^ for a minimum of 5 days with daily media changes.

### Drug treatments

5α‐Dihydrotestosterone (DHT; Sigma‐Aldrich, Dorset, England) was dissolved in ethanol at 4.5 nM, and equivalent vehicle was added to controls and filtered using a 0.2 μm filter, 1 h prior to the start of an experiment. An antagonist was used, Flutamide at 3 μM (Tocris, Abingdon, England).

Cells were starved in HBSS (Sigma‐Aldrich) for a total of 150 min. Where indicated, amino acid supplement of 1.1% amino acids were added and FBS increased to 20% for 150 min to act as overfed.

### Cloning SNAT2‐eGFP

The gene sequence NM_175121 for SNAT2 (see Supporting Information, *Figure* S1A) was cloned into the vector pcDNA3.1‐C‐eGFP by Genscript (Leiden, Netherlands), hereafter identified as SNAT2‐eGFP. Thereafter, 10 ng of plasmid was heat shocked into *E. coli* strain DH5α (Invitrogen, Thermo Fisher, UK) in a small amount of SOC media. The transformed *E. coli* were grown in antibiotic free LB‐broth for 6 h and then a small amount spread on agar plates containing 100 mg/mL ampicillin (Sigma‐Aldrich) and grown overnight at 37°C. A selection of colonies were picked the next day and grown in separate flasks of LB‐broth containing 100 mg/mL ampicillin for 16 h on a shaking incubator at 37°C at 800 rpm. Thereafter, the LB was centrifuged in universal tubes to pellet the *E. coli*, the plasmid DNA was extracted from the cells using standard methods (Plasmid mini‐prep, Macherey Nagel, Fisher Scientific, Leicestershire, England). The final plasmid DNA was checked for quality by a spectrophotometer and a small amount run on a 2% agarose gel (Sigma‐Aldrich) after cutting at selected restriction sites (using standard methods), see Supporting Information, *Figure* S1B.

### Transfection

Myoblast cells were grown on glass cover slips coated with laminin (50 μg/cm^2^). In brief, 0.5 μg per well of plasmid SNAT2‐eGFP DNA was transfected by Lipofectamine 2000 (Thermo Fisher) into cells by standard techniques. Briefly, cells were grown on coverslips till approximately 70–80% confluent and then transfected with Lipofectamine for 24 h and then washed with media. The coverslips were then used as indicated for additional experiments. Other transfections were carried out in an identical manner using 6‐well plates or T25 flasks. Transfection efficiency was determined from four separate transfections, where at least three coverslips per transfection were counted for the number of GFP positive cells. This analysis indicated 16 ± 1.3% (range 13–19.3%) GFP positive cells. Transfections that failed to attain this approximate efficiency range of transfection were not used. Control cells were transfected identically using vehicle alone.

### Immunocytochemistry

Cells were fixed with 4% PFA and permeabilized by exposure to 2% NP40/2% methanol, primary antibody for SNAT2 (SNAT2 H‐60 Sc‐166,366 mouse monoclonal, Santa Cruz, USA) was used at 1:50 in 1% BSA/TBST, secondary antibodies were applied in 2% BSA/TBST for 1 h at room temperature by standard procedures and stained with DAPI. Control coverslips had no primary antibody.

Cells were imaged on a Zeiss Imager M2 Apotome microscope with a 63×, 1.4 NA oil‐immersion objective, and images were captured using AxioVision V.4.9.1.0 (Carl Zeiss Microscopy, Germany).

### Western blotting

Briefly, cells lysed in RIPA buffer (150 mM sodium chloride, 1.0% NP‐40, 0.5% sodium deoxycholate, 0.1% SDS, 50 mM Tris, pH 8.0) containing 10 mg/10 mL protease inhibitors (Roche, Switzerland) and 100 μL/10 mL phosphatase inhibitors (Calbiochem, Nottingham) generated protein lysates (in standard 2× Laemmli buffer), which were separated by standard gel SDS‐PAGE electrophoresis and transferred onto nitrocellulose membranes (BioRad, UK). In some cases, membranes were cut at certain target kDa weights to allow different portions of a membrane to be exposed to a different single primary antibody (see Supporting Information, *Figure* S2). Membranes were blocked for non‐specific antibody binding for 2 h using either 5% milk or 3% BSA in TBST (Tween 0.037%) and immunoblotted for the expression of SNAT2 (Sc‐166366; Santa Cruz Biotechnology, USA), GFP (Abcam, Cambridge, UK), GAPDH (Abcam), and actin (Sigma), whilst HRP conjugated secondary antibodies were used to visualize protein using SuperSignal WestPico chemiluminescence substrate (Perbio Science UK Ltd, Cramlington and Northumberland, UK). Densitometry analysis of a protein of interest was normalized to that samples actin/GAPDH, and a minimum of three different replicates were undertaken.

### Determining SNAT2 and LAT2 transport in C2C12 cells

C2C12 cell uptake of 68.3 μM C^14^‐α‐methylaminoisobutyric acid (0.8 μCi/mL; C^14^‐MeAIB) or 2 mM C^14^‐isoleucine (3.46 μCi/mL; C^14^‐Iso, PerkinElmer, Bucks, England) was used to quantify SNAT2 and LAT2 transport, respectively. Radio‐isotopes were added at the start of each 1 h experiment.

After 1 h, cells were washed and put into 5 mL glass tubes, centrifuged at 800 *g*. The pellet was re‐suspended in 100 μL of lysis buffer and put on ice for 20 min, sonicated with a needle sonicator until the pellet was dispersed and then centrifuged at 13 000 *g* for 10 min. The pellet was discarded, and the amino acids in total cell lysate supernatant were counted by scintillation counting.

Radioactivity in each lysate was determined using a liquid scintillation counter (Tri‐Carb 2250 CA, Canberra‐Packard) by standard procedures. Each experiment was run a minimum of three times, using different cell culture preparations for each replicate.

### Statistical analysis

Data were analysed to determine normality using the Shapiro–Wilk test; the *P* value in all cases indicated that the data sets had normal distributions. Data are presented as mean ± SEM for each experiment. Statistical analysis for potential differences between groups was determined using analysis of variance (ANOVA) followed by the *post hoc* LSD test. Single comparisons between two experimental conditions were determined by unpaired Student's *t*‐test. Data were analysed using SPSS V.22, and *P* values <0.05 were considered statistically significant.

## Results

### SNAT2 expression in C2C12 myoblasts and myotubes

**Figure 1:**
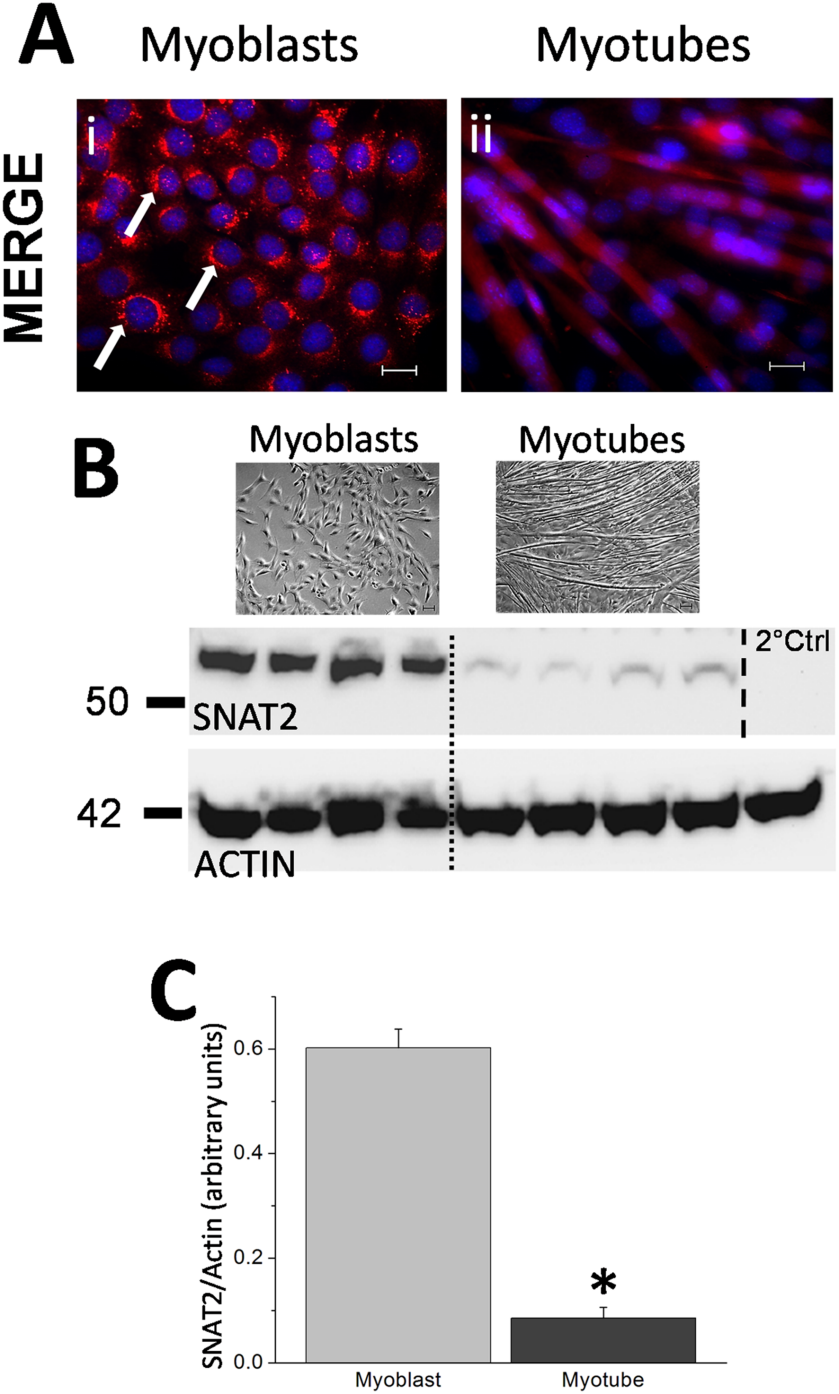
(A) Immunocytochemistry of *de novo* SNAT2 expression in C2C12 myoblast and myotube cells. C2C12 myoblast cells (i) were grown on cover slips for several days; thereafter, cells were probed using an antibody for SNAT2 expression (red), and DAPI (blue) was applied to show the cells nuclei. Additional cover slips of myoblast cells were differentiated into myotubes (ii) for 5 days and again probed for SNAT2 and DAPI. Panel (A‐i) indicates numerous perinuclear puncta indicating SNAT2 expression in myoblasts, and several examples of puncta are indicated by white arrows. Panel (A‐ii) shows SNAT2 expression in myotubes indicating cytoplasmic expression and little evidence of puncta. Scale 20 μm. (B) Example SNAT2 western blot of myoblast and terminally differentiated myotube cells. Example bright field pictures (top) of cells (scale bar 20 μm) are shown above the western blot that shows four separate repeats (middle) from the indicated myoblast and myotube cells (separated by a dotted line for clarity) when probed for SNAT2 expression. The bottom panel shows house keeper protein expression for actin that was used to validate loading between the sample groups. A secondary antibody control step was undertaken where the SNAT2 primary antibody was not used although it was still exposed to the HRP‐conjugated secondary antibody and shows secondary antibody specificity (where the dashed line indicates the membrane being cut and no primary antibody was applied). The primary antibody for the house keeper actin was exposed to all parts of the blot. (C) Histogram of densitometry analysis from the western blot (B) showing a significant reduction in SNAT2 expression (indicated by an asterisk) in myotubes (dark grey) versus myoblasts (light grey, *n* = 4 per group, Student's *t*‐test, *P* < 0.05, data presented as mean ± SEM).

SNAT2 expression was investigated in low passage myoblast (*Figure* 1B, top left) cells or in cells differentiated into myotubes for 5 days (*Figure* 1B, top right). Typical results show strong perinuclear puncta of SNAT2 staining in all myoblasts (shown in red, *Figure* 1A indicated by arrows), whereas myotubes showed more diffuse cytoplasmic fluorescence. Western blotting for the expression of SNAT2 demonstrated a significant difference between myoblast and myotube SNAT2 expression (*Figure* 1B; see Supporting Information, *Figure* S2, top), the proliferating myoblast cell expression of SNAT2 was approximately 6.5× more abundant than that found in terminally differentiated myotube cells (see *Figure* 1C, *n* = 3 per group, Student's *t*‐test). Myoblasts are proliferating cells responsible for repairing damaged myotubes or after merging can generate new myotubes; therefore, subsequent experiments were undertaken using myoblasts only.

### Effect of starvation or excess amino acid supplementation upon the *de novo* expression of SNAT2 in C2C12 myoblasts

**Figure 2:**
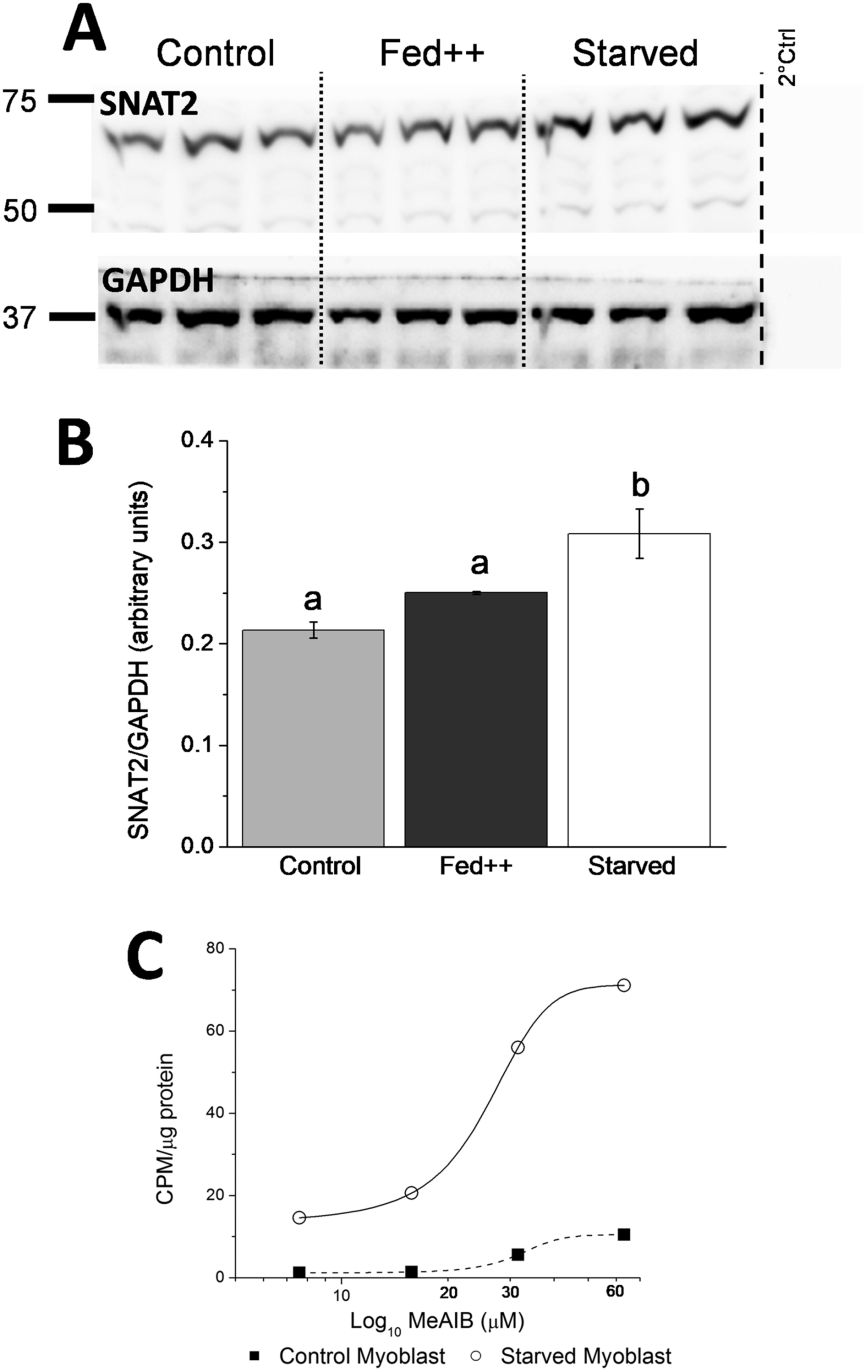
(A) Western blot of myoblast cells probed for expression of SNAT2 (top) and loading control GAPDH (bottom), three separate repeats per treatment group of; control, fed++ (given increased amino acid/FBS supplement) and starved (HBSS) were undertaken, where treatments were applied for 150 min, and thereafter, western blot sample preparation was immediately performed. Dotted lines show different groups for clarity, whilst the dashed line shows where the blot was cut to exclude primary antibody application to determine secondary antibody specificity. (B) Densitometry analysis of each treatment group in the western blot (A) showed a significant increase in SNAT2 expression in only the starved group versus either control or fed++, where b > a, *P* < 0.05 (*n* = 3 per group, one‐way ANOVA, data presented as mean ± SEM). (C) Dose–response curve plot showing fed myoblast control cells (solid square) and 150 min starved myoblast (open circle) C2C12 cells C^14^‐MeAIB transport versus increasing C^14^‐MeAIB concentrations, results indicate enhanced transport at all given concentrations in response to starvation. Curves fitted using a Boltzmann plot (Origin, Origin Lab Cooperation, USA).

To investigate factors that control SNAT2 expression in C2C12 myoblasts, cells were cultured with additional amino acids (Fed++) or in HBSS media with no amino acids to induce starvation. After 150 min, treated cells were harvested, and proteins were analysed by western blotting (*Figure* 2A; see Supporting Information, *Figure* S2, bottom). There was no significant effect upon SNAT2 expression in the Fed++ group when additional excess amino acids were applied, whereas there was a significant increase in SNAT2 expression following starvation (*Figure* 2B). This indicated that SNAT2 recruitment was induced in a low amino acid environment.

Myoblast cells were then grown in standard media (*Figure* 2C) or in HBSS to induce starvation, and after 90 min starvation, the cells were exposed to differing concentrations of C^14^‐MeAIB for one additional hour (total 150 min treatment) to generate a dose–response curve. *Figure* 2C indicated that starved cells transported more C^14^‐MeAIB from the media to inside the cell than control fed myoblasts. The EC_50_ for fed versus starved was 32 and 26 μm C^14^‐MeAIB respectively showing greater amino acid uptake by SNAT2 in a starved environment.

### Expression of SNAT2‐eGFP in myoblasts

**Figure 3:**
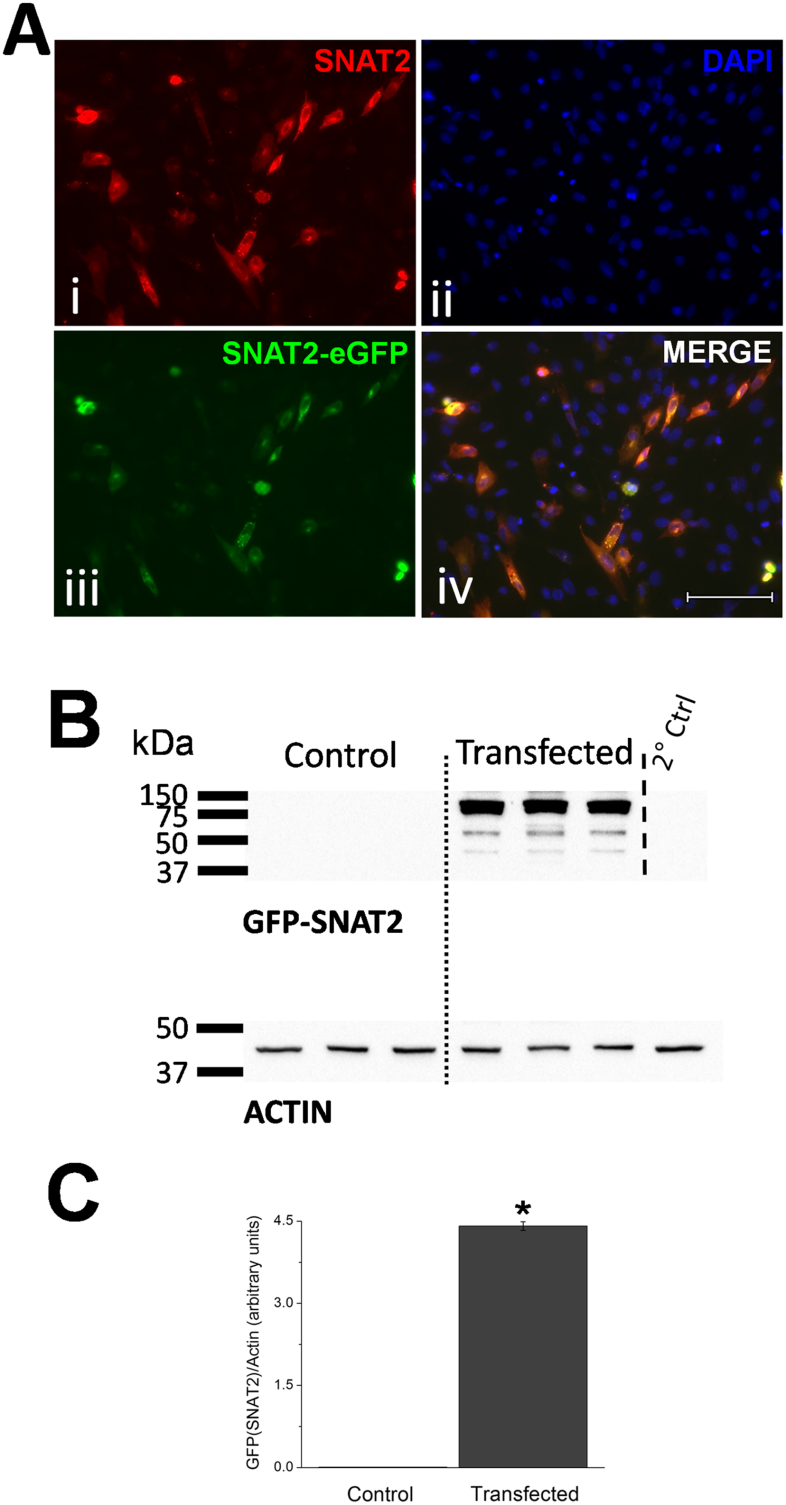
(A) Fluorescent images of transfected C2C12 myoblast cells. Immunocytochemistry shows SNAT2 (i, red indicated combined *de novo* and transfected SNAT2), nuclei staining (ii, blue, DAPI), plasmid transfected SNAT2 (iii, green, SNAT2‐eGFP), and a merged image (iv) showing co‐localisation of *de novo* SNAT2 and SNAT2‐eGFP overexpression. Typical transfection rate of cells was approximately 16%. Scale 100 μm. (B) Western blot of myoblast cells probed using a polyclonal antibody for GFP (top) and actin (bottom) used as a loading control, showing control (untransfected) and SNAT2‐eGFP transfected cell protein expression of GFP at approximately 83 kDa (i.e. SNAT2 + GFP fusion protein weight combined), *n* = 3 separate repeats per group separated by a dotted line for clarity. (C) Densitometry analysis of the western blot (B) showing a significant difference in the transfected group expression of GFP associated SNAT2 24 h after SNAT2‐eGFP plasmid transfection (Student's *t*‐test, *n* = 3, *P* < 0.05, data presented as mean ± SEM).

SNAT2 cDNA was synthesized commercially, and the sequence is given in the Supporting Information, *Figure* S1A. It was cloned into pcDNA3.1‐eGFP and characterized by restriction digest (see Supporting Information, *Figure* S1B). The SNAT2‐eGFP DNA was transfected into myoblasts, and the cells were either immunostained for SNAT2 or imaged by direct fluorescence. The immunostaining corresponded to the direct GFP fluorescence indicating that the signal was specific for SNAT2 (*Figure* 3A, iii), and SNAT2 expression was confirmed by western blotting using a polyclonal antibody to the GFP tag (see *Figure* 3B; see Supporting Information, *Figure* S2) and showed a band for the fusion protein at approximately 83 kDa. Densitometry analysis showed significant expression of GFP compared with untransfected control cells.

**Figure 4:**
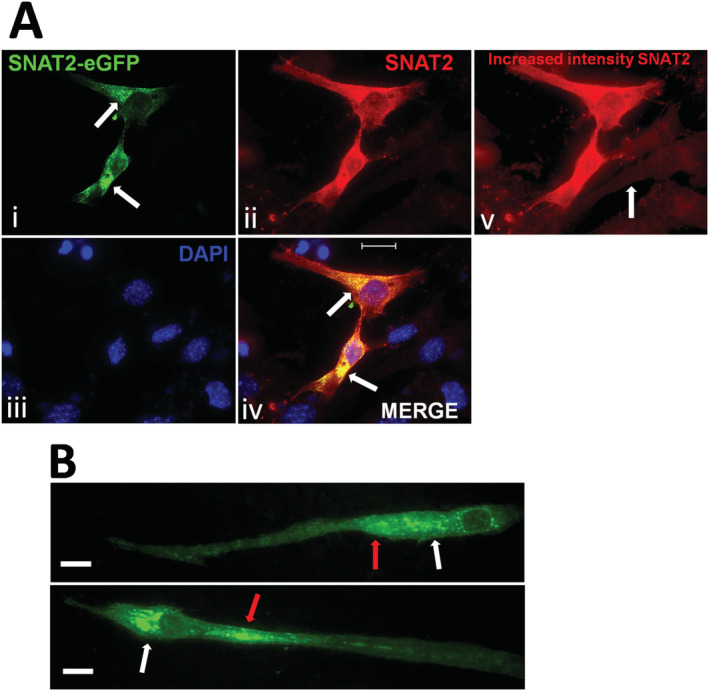
(A) Fluorescent images of SNAT2‐eGFP transfected myoblast cells showing, (i) SNAT2‐eGFP transfected (white arrows indicate perinuclear puncta of SNAT2‐eGFP, whilst SNAT2‐eGFP also extended to other cellular regions without puncta), (ii) immunocytochemistry from *de novo* SNAT2 (includes SNAT2‐eGFP protein), (iii) nuclei stained using DAPI, (iv) merged image (i–iv, white arrows indicate perinuclear puncta of SNAT2‐eGFP and co‐localisation of *de novo* SNAT2 and SNAT2‐eGFP). Image (v) shows an increased exposure fluorescent image using image (ii) to show transfected cells overexpressing SNAT2 and *de novo* SNAT2 expression from nearby untransfected cells (an untransfected example is indicated by the white arrow) both being targeted by a primary antibody to SNAT2. Scale 20 μm. (B) Fluorescent image, of two examples, of myoblast cells previously transfected with the plasmid for SNAT2‐eGFP and thereafter differentiated into myotubes (pictured) for 5 days showing retention of SNAT2‐eGFP. White arrows indicate SNAT2 puncta in close proximity to the nuclei with additional SNAT2 puncta being more cytoplasmic and distant (red arrows). Further cytoplasmic fluorescence of SNAT2‐eGFP extended along the length of the myotubes. Scale 25 μm.

SNAT2‐eGFP expression (*Figure* 4A, i) was punctate and peri‐nuclear with some cytoplasmic fluorescence, whereas endogenous SNAT2 (*Figure* 4A, ii) was predominantly cytoplasmic with some punctate fluorescence. Untransfected cells shown in *Figure* 4A(v) showed low levels of endogenous SNAT2 expression throughout the cytoplasm. Myoblasts were successfully differentiated for 5 days into myotubes (*Figure* 4B), and these myotubes showed some peri‐nuclear and predominantly cytoplasmic punctate fluorescence indicated by white and red arrows, respectively. Overexpressed SNAT2‐eGFP was also diffusely observed throughout the myotube cytoplasm.

### SNAT2 and SNAT2‐eGFP functional transport of the isotope C^14^‐MeAIB

**Figure 5:**
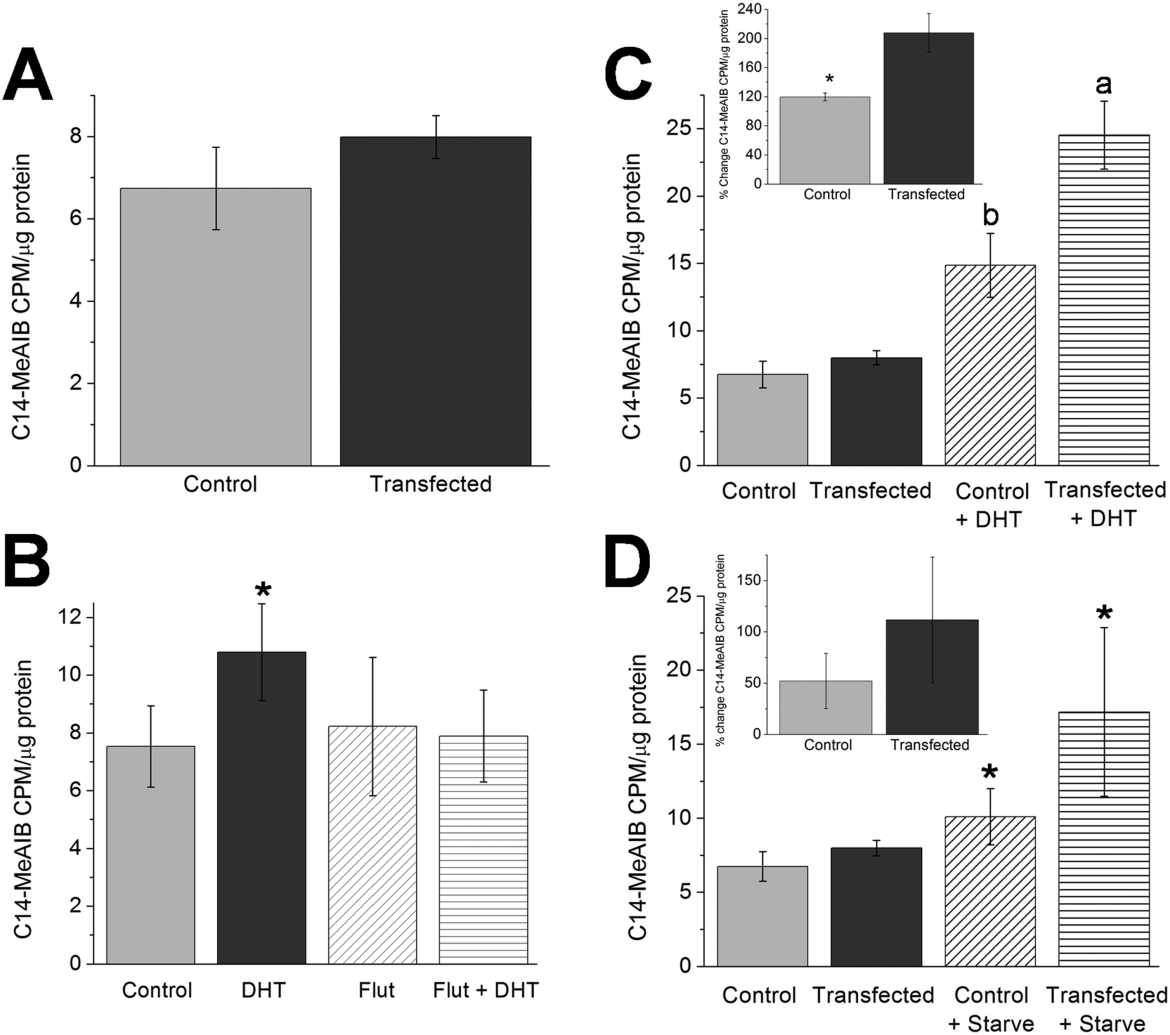
(A) Myoblast cells were exposed to C^14^‐MeAIB for 1 h, and thereafter, uptake of the C^14^‐MeAIB was measured using scintillation counting; cells were either not transfected control (light grey) or transfected with SNAT2‐eGFP (dark grey). A small but not significant increase in C^14^‐MeAIB transport was recorded in the transfected myoblasts (Student's *t*‐test, *n* = 3 both groups, data presented as mean ± SEM). (B) The effect of the hormone DHT was investigated upon untransfected myoblast cells that were treated as control, DHT (4.5 nM) or pre‐treated with the androgen receptor antagonist flutamide (3 μM) alone or for 1 h prior to an identical DHT exposure. DHT treatment alone resulted in a significant (*) 46 ± 10% increase in C^14^‐MeAIB transport. Flutamide treatment irrespective of DHT exposure was not different from control (*n* = 4, one‐way ANOVA, *P* < 0.05, data presented as mean ± SEM). (C) Myoblast cells were either untreated or exposed to 4.5 nM DHT for 1 h and thereafter exposed to C^14^‐MeAIB for one additional hour, and the uptake of C^14^‐MeAIB was measured. This was carried out to control (not transfected) and SNAT2‐eGFP transfected groups. DHT significantly increased C^14^‐MeAIB transport in untransfected and transfected myoblasts (*n* = 3, one‐way ANOVA where a > b, *P* < 0.05). Inset histogram shows the percentage change in the DHT stimulated transport of C^14^‐MeAIB calculated from the main figure. Transfected myoblasts showed a significant enhancement of C^14^‐MeAIB transport (207.7 ± 26.6%, dark grey) versus control untransfected cells (119.8 ± 5%, light grey, *n* = 3, *P* < 0.03, Student's *t*‐test, data presented as mean ± SEM). (D) Myoblast cells were either fed or starved for 150 min and then exposed to C^14^‐MeAIB for 1 h and uptake of C^14^‐MeAIB measured in control (not transfected) and SNAT2‐eGFP transfected groups. Starvation significantly increased C^14^‐MeAIB transport in untransfected and transfected myoblasts (*n* = 3, one‐way ANOVA, *P* < 0.05) versus their not starved controls. Inset histogram reports the percentage change in starvation induced transport of C^14^‐MeAIB shown in the main figure, and no significant increase in C^14^‐MeAIB transport after transfection (111.6 ± 61.3%, dark grey) was found versus control untransfected cells (52.2 ± 27%, light grey, *n* = 3, Student's *t*‐test, *P* > 0.05, data presented as mean ± SEM).

Amino acid transport was measured in cells expressing SNAT2‐eGFP using an amino acid analogue C^14^‐MeAIB, a substrate for SNAT2 transport. C^14^‐MeAIB transport (in full media) in control and SNAT2 cDNA transfected C2C12 cells showed a small but non‐significant increase (16.2 ± 9%, *Figure* 5A) in C^14^‐MeAIB transport (Student's *t*‐test, *n* = 3, *P* = 0.3). In control cells, transport of C^14^‐MeAIB was measured (*Figure* 5B) in response to DHT (4.5 nM) or flutamide (3 μM) alone or flutamide followed by DHT treatment to investigate the androgen receptor hormone response of endogenous SNAT2 function, because DHT has been previously shown in primary tissue to significantly increase amino acid transport by SNAT2.^18^ Our results show a significant increase in C^14^‐MeAIB transport after treatment with DHT alone (46 ± 10%, *n* = 4, one‐way ANOVA). This was entirely blocked by pre‐treatment with flutamide, an androgen receptor antagonist, irrespective of whether it was followed by DHT, and this confirmed *de novo* SNAT2 sensitivity to the hormone DHT via the androgen receptor.

The effect of DHT exposure (*Figure* 5C) in C2C12 cells transfected with the SNAT2‐eGFP cDNA plasmid was investigated. There was an increase in C^14^‐MeAIB transport in both untransfected and transfected cells following DHT treatment. The enhancement in the DHT response after transfection was (207.7 ± 26.6%) significant compared with DHT treatment of control cells (119.8 ± 5%, *n* = 3, inset histogram) and clearly demonstrated physiological sensitivity of SNAT2‐eGFP protein to the hormone DHT.

When cells were starved (*Figure* 5D) of amino acids with HBSS for 150 min, there was a significant increase in the transport of C^14^‐MeAIB in both control and transfected cells that was again enhanced by SNAT2‐eGFP transfection (inset histogram) although that enhancement was not different from control.

### Does SNAT2‐eGFP transfection have any effect on LAT2 transport of C^14^‐isoleucine?

**Figure 6:**
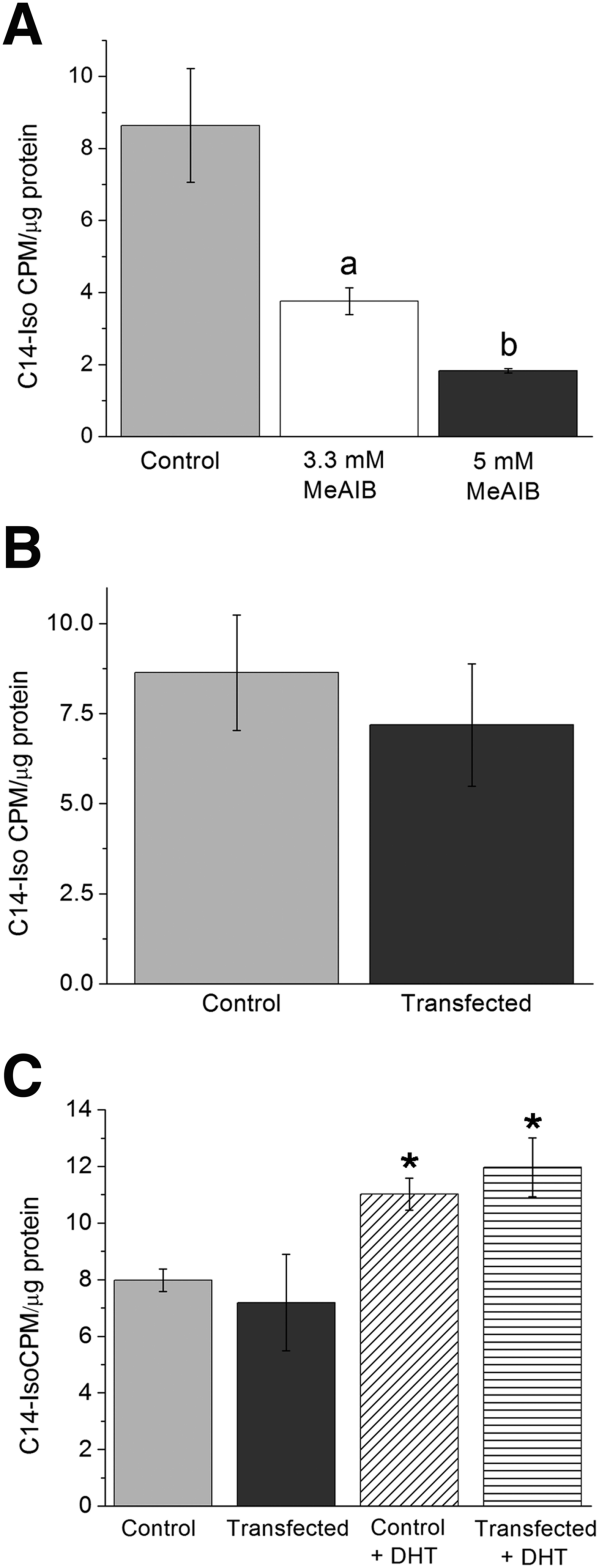
Impact of SNAT2 transport function upon LAT2 transport of C^14^‐isoleucine. (A) Histogram showing untransfected myoblast cells, where treatments were control or cells treated with 3.3 mM or 5 mM MeAIB (not C^14^) respectively to block SNAT2. Blocking SNAT2 with high dose MeAIB reduced C^14^‐isoleucine transport by LAT2 (52.8 ± 5% and 77 ± 2.5%, respectively), significantly so at both concentrations used (*n* = 3 per group, one‐way ANOVA, *P* < 0.05, data presented as mean ± SEM). (B) Myoblast cells were exposed to C^14^‐Iso for 1 h, and thereafter, uptake of the C^14^‐Iso was measured, and cells were either not transfected control (light grey) or transfected with SNAT2‐eGFP (dark grey). A small but not significant (Student's *t*‐test, *P* > 0.05) decrease in C^14^‐Iso transport was recorded in the transfected myoblasts (*n* = 3 both groups, data presented as mean ± SEM). (C) Histogram showing C^14^‐isoleucine transport in control (not transfected) and SNAT2‐eGFP transfected C2C12 cells that were either untreated or treated with 4.5 nM DHT for 1 h. Treatment with DHT significantly increased C^14^‐isoleucine transport to a similar level irrespective of transfection with SNAT2‐eGFP (*n* = 3, one‐way ANOVA, *P* < 0.05, data presented as mean ± SEM).

SNAT2‐eGFP expression may affect the ability of LAT2 to transport C^14^‐Iso depending on the intracellular availability of small amino acids. *Figure* 6A demonstrated that when SNAT2 transport of amino acids was blocked by higher doses of MeAIB, there was a significant dose dependant decline in C^14^‐Iso transport in control C2C12 cells. This was likely due to reduced intracellular small amino acid availability for the LAT2 anti‐porter and importantly confirmed a mechanistic link between SNAT2 transport and LAT2 function. C2C12 cells either control or transfected with SNAT2‐eGFP showed no difference in C^14^‐Iso transport by LAT2 in full media (*Figure* 6B), indicating no shortage of small amino acid anti‐porter substrates to facilitate larger amino acid transport by LAT2. Control and C2C12 cells transfected with SNAT2‐eGFP (*Figure* 6C) had a similar significant (one‐way ANOVA) increase in DHT stimulated C^14^‐Iso transport, that was not different between control versus transfected cells.

## Discussion

In this study, we used the C2C12 cell line to express the SNAT2 amino acid transporter and investigated its function in small neutral amino acid transport.^24^ Previous studies have reported from primary tissue a decline in SNAT2 expression with ageing; for example, in both slow and particularly fast twitch skeletal muscle fibre types, the authors argue that a decline in SNAT2 (and LAT2) was a contributor to the pathology of sarcopenia.^18^ They further reported a reversal of transporter loss after DHT treatment. We have built upon this observation using the C2C12 cell line to examine if SNAT2 overexpression would yield functional amino acid transporters that were responsive to physiological stimuli. Importantly we then investigated whether there was a secondary positive effect upon LAT2 anti‐porter transport function to determine a mechanistic link between SNAT2 and LAT2. We chose to investigate SNAT2 from the SLC38 gene family as it is the only amino acid transporter (with LAT2) consistently found in skeletal muscle,^15^ and importantly, SNAT2 has the ability to transport a non‐metabolizable substrate; methyl‐aminoisobutyrate (MeAIB) used in this study and by others^25^ to follow SNAT2 transporter function.

### SNAT2 expression in myoblasts and myotubes

The overexpression of SNAT2 in myoblasts was maintained through to mature myotubes (*Figure* 4B) after differentiation. This has the potential for its use as an epigenetic intervention in differentiated cells. Skeletal muscle has a robust innate ability to repair after injury through the presence of satellite cells, whilst muscle fibres are post mitotic having no capacity to divide or initiate any regeneration.^26^ We utilized myoblasts as this cell type is crucial to muscle repair or formation of new muscle fibres and is relevant as other studies have used C2C12 cells as a myoblast therapy for systemic delivery of recombinant proteins.^27^ Here, the C21C12 cells could be utilized in a similar way to deliver SNAT2 amino acid transporters to compromised muscle types to potentially aid recovery from trauma or slow sarcopenia through SNAT2 dependent amino acid transport. The cellular distribution of SNAT2 after differentiation into myotubes changed from being predominantly perinuclear in myoblasts to being more diffuse and cytoplasmic, with less puncta evident in perinuclear areas in the myotubes. This implied a different internal distribution of SNAT2‐eGFP in myotubes compared with myoblasts. Endogenous SNAT2 expression in mature myotubes was 6× lower than detected in myoblasts (*Figure* 1B,C). Logically, the expression difference of endogenous SNAT2 demonstrates demand for amino acids of growing proliferating myoblast cells, whereas terminally differentiated myotubes express less amino acid transporters for cell maintenance purposes only as proliferation had ceased. This might argue a more abundant diffuse distribution of SNAT2 along a fibre length could contribute to viability.

### Effects of stimuli upon SNAT2

SNAT2 expression is reported to be adaptive, and amino acid removal has been reported to increase SNAT2 protein stability and SNAT2 gene transcription.^28^ Bed rest has been reported to decrease amino acid transporter expression in older people,^29^ and the ability of transporters to function has also been reported to be responsive to amino acid concentration levels.^30^ It was argued that an acute phase over minutes could recruit SNAT2 from internal pools^19^ in response to amino acid deprivation. This supports our observation of increased *de novo* SNAT2 expression in response to a period of starvation (*Figure* 2A,B) and that SNAT2‐eGFP could also be recruited in a similar way to contribute to transporter function in response to stimulus treatments of, for example, DHT (*Figure* 5C,D). Importantly, other works observed increased Vmax (maximal transport) rather than Km^31^, ^32^, ^33^ arguing an up‐regulation in transport by SNAT2 could be solely accounted for by the quantity of functioning transporters on the plasma membrane. The mechanism behind the adaptive response of SNAT2 availability is unknown although the time course is reminiscent of insulin upon GLUT4 recruitment from internal vesicular pools.^34^

Furthermore, as SNAT2 is a system A transporter, it is reported to be sensitive to trans‐inhibition, where accumulation of its substrate causes inhibition of the transporter^35^ and thus can function to control the intracellular amino acid cytoplasmic concentration.^16^ *Figure* 2C supported this observation as untransfected fed myoblasts transported less C^14^‐MeAIB at each concentration investigated as compared to starved myoblasts, indicating trans‐inhibition and a move of SNAT2 to internal storage sites when exposed to sufficient amino acid substrates. An observation further supported by *Figure* 2A,B, as no change in SNAT2 expression upon being fed extra amino acids was seen and argued full growth media was sufficient to saturate the amino acid sensing mechanism of SNAT2. The mechanism of SNAT2 sensing amino acid levels is not clearly understood, it has been shown that amino acid starvation induced up‐regulation of SNAT2 is reduced by PI3K and JNK inhibitors.^33^ Furthermore, transfection of SNAT2‐eGFP only modestly raised the transport of C^14^‐MeAIB (16%, *Figure* 5A) when compared with untransfected cells, and this important observation indicated that SNAT2‐eGFP was functional in regard of sensing intracellular amino acid levels as increased transporter expression did not automatically correlate to increased transport; additional stimuli were required, such as DHT, to activate SNAT2‐eGFP transport of C^14^‐MeAIB.

### Are transfected SNAT2 functional?

In this study, it is likely that transfection of SNAT2‐eGFP in only 16 ± 1.3% of cells, generated transporters that were localized to internal storage sites.^19^ The significantly greater transport of C^14^‐MeAIB in response to a DHT treatment after SNAT2‐eGFP transfection than found in control myoblasts in response to an identical DHT (see *Figure* 5C, and inset) stimulus argues that the SNAT2‐eGFP was functional and able to be recruited, an observation also supported by the trend of increased C^14^‐MeAIB transport in response to starvation (*Figure* 5D) although that response was more variable. *In vivo* it is recommended that ageing humans eat a high quality protein source shortly after resistance exercise, as a stimulus, to maximize any anabolic response^36^ to offset ageing anabolic blunting. Hawkins *et al*.^37^ reported increased DHT production in men over 40 years of age after exercise.

### Is LAT2 function affected by SNAT2 transport of amino acids?

We investigated whether SNAT2 expression might have a positive effect upon transport of branched chain amino acids by LAT2. This could potentially contribute to conditions where amino acid transport may be compromised, for example, sustained bed rest and sarcopenia. We reported that increasing concentrations of the SNAT2 blocker MeAIB (3.3 and 5 mM) significantly reduced the transport by LAT2 of isoleucine and confirmed the crucial importance of SNAT2 transported substrates upon the function of LAT2 transport, confirming studies by Hatanaka *et al*.^38^ SNAT2 transport of small amino acids may be an important target to maintain LAT2 function during muscle stress such as ageing. However, we reported no significant impact upon LAT2 transport following SNAT2 transfection (*Figure* 6B) or a difference in response to DHT treatment (*Figure* 6C), indicating that the availability of small amino acids in this case was not limiting LAT2 transport. This argues that increasing intracellular LAT2 anti‐porter substrate levels by increasing SNAT2 transporter availability is not a feasible method to increase LAT2 activity directly under normal conditions. Additional work is needed to accurately define the impact of SNAT2 transport upon LAT2 function in muscle undergoing a period of pathology or stress. It is known the nutrient signal in aged muscle is not sensed or transduced as well as in young muscle given an identical nutrient stimulus, such as sensing a flooding dose of leucine^36^ which resulted in a lower protein synthesis response from aged muscle.

## Conclusions

These data show that expression of endogenous SNAT2 protein changes as myoblasts differentiate into myotubes. SNAT2 expression increased in response to starvation whilst transport by SNAT2 was also increased after a DHT treatment. Additionally, transport of C^14^‐MeAIB by SNAT2‐eGFP and its responsiveness to DHT and starvation was greater than that of endogenous SNAT2. Together, these results indicate that if SNAT2 lost during pathology can be restored and functional, it would be able to increase amino acid concentrations. However, overexpressed SNAT2 had no impact on LAT2 transport of isoleucine under basal conditions, indicating that intracellular concentrations of small amino acids were not a limiting factor. The increase in C^14^‐Iso transport to a DHT treatment was similar, irrespective of transfection, and indicated that LAT2 was responsive to DHT although was not limited by anti‐porter substrate availability provided by SNAT2.

Overall, these data show that SNAT2 may be a target for epigenetic modification to offset altered amino acid transport during pathological situations or to enhance amino acid transport after other stimuli such as exercise.^12^

## Data availability statement

The data that support the findings of this study are presented in full form within the supporting information, and other data are available on request from the corresponding author.

## Conflict of interest

None declared.

## Supporting information


**Figure S1:** **(A)** Full nucleotide sequence coding amino acid transporter SNAT2 (Slc38a2, NM_175121) that yields a 506 amino acid protein (56 kDa, NP_780330). **(B)** Example restriction digest of pcDNA3.1‐C‐SNAT2eGFP (SNAT2‐eGFP) plasmid showing, ladder, uncut, single cut using XBal, double cut using HindIII and NotI restriction enzymes. Uncut plasmid runs to approximately 4,400 bases, single digest using Xbal linearises the plasmid with a product of 7,650 bases whilst a double cut generates two fragments of approximately 1,515 bases, being SNAT2, and the remainder 6,143 bases being the empty cloning vector.

**Figure S2:** **Blot Figure 1B**: Shown are the Western blots from main text Figure 1B. The membrane was cut into two pieces to allow separate primary antibody application to each blot piece. SNAT2 primary antibody was applied to the top blot portion except to the final 2°Ctrl lane (indicated by dashed line) to determine any non specific secondary antibody binding. Primary antibody to actin was applied to the bottom blot across the whole membrane to act as a loading control and to validate protein had been loaded in that 2°Ctrl lane i.e. lane not exposed to SNAT2 primary antibody (above). Dotted lines indicate different sample groups for clarity. **Blot Figure 2A**: Shown are the Western blots from main text Figure 2A. The membrane was cut into two pieces to allow separate primary antibody application to each blot piece. SNAT2 primary antibody was applied to the top blot portion whilst a primary antibody to GAPDH was applied to the bottom blot except to the final 2°Ctrl lane (indicated by dashed line) to allow non specific secondary antibody binding to be determined. Dotted lines indicate different sample groups for clarity.

**Figure S2.** **Blot Figure 3B**: Shown are the Western blots from main text Figure 3B. The entire membrane was exposed to a GFP polyclonal primary antibody (top blot) except the final 2°Ctrl lane (indicated by dashed line) which was cut away and thus allowed non specific secondary antibody binding to be determined. Primary antibody to actin (bottom blot) was thereafter applied to all of the membrane and validated protein had been loaded in the 2°Ctrl lane not exposed to the GFP primary antibody (above) and also acted as a loading control. Dotted lines indicate different sample groups for clarity.
